# Prevalence of 7 sexually transmitted organisms by multiplex real-time PCR in Fallopian tube specimens collected from Saudi women with and without ectopic pregnancy

**DOI:** 10.1186/s12879-015-1313-1

**Published:** 2015-12-15

**Authors:** Ahmed Mohamed Ashshi, Sarah Abdullah Batwa, Seham Yahia Kutbi, Faizah Ahmed Malibary, Mohamed Batwa, Bassem Refaat

**Affiliations:** Laboratory Medicine Department, Faculty of Applied Medical Sciences, Umm Al-Qura University, Al Abdeyah, Makkah, PO Box 7607, KSA; Obstertics and Gynaecology Department, Maternity and Children Hospital, Al-Aziziyah, Jeddah, KSA; Al-Thager General Hospital, Jeddah, KSA

**Keywords:** *Chlamydia trachomatis*, *Mycoplasma genitalium*, Herpes simplex virus, Ectopic pregnancy, Multiplex PCR, Saudi Arabia

## Abstract

**Background:**

Ectopic pregnancy (EP) is associated with maternal morbidity and occasionally mortality during the first trimester. A history of sexually transmitted infection (STI) and pelvic inflammatory disease have been implicated as major risk factors for EP. Our aim was to measure the prevalence of *Chlamydia trachomatis* (CT), *Neisseria gonorrhoeae*, *Mycoplasma genitalium* (MG), *Ureaplasma parvum/urealyticum*, *Gardnerella vaginalis*, *Trichomonas vaginalis* and herpes simplex virus (HSV)-1&2 in Fallopian tubes collected from EP and the results were compared with those obtained from total abdominal hysterectomy (TAH) and tubal ligation.

**Methods:**

This was a prospective case–control study and tubal samples were collected from 135 Saudi women recruited from 3 centres in the Western region as follow: 84 EPs, 20 TAH and 31 tubal ligations. Multiplex TaqMan PCR was performed using an IVD CE kit for the simultaneous detection of candidate pathogens following DNA extraction.

**Results:**

Infections were detected in 31.8 % of the 135 participants either as single (11.1 %) or co-infections (20.7 %) and the frequencies were significantly higher in EP (42.85 %) compared with control (13.72 %). The rates of CT (27.4 %; *P* = 0.001); MG (20.2 %; *P* = 0.009) and HSV-1/2 (21.4 %; *P* = 0.01) were significantly higher in EP. No significant difference between the study groups was observed for the other pathogens (*P* > 0.05). Binary logistic regression also showed that infection with ≥ 2 pathogens (OR 4.9; 95 % CI: 2.2 – 11.6; *P* = 0.006), CT (OR 3.07; 95 % CI: 1.3 – 12.3; *P* = 0.002), MG (OR 2.3; 95 % CI: 1.1 – 8.6; *P* = 0.03) and HSV-1/2 (OR 1.7; 95 % CI: 0.75 – 5.7; *P* = 0.004) were associated with a significantly higher risk of developing EP.

**Conclusions:**

STIs are frequent in the upper genital tract of Saudi women during the reproductive age and, CT, MG and HSV-1/2 were more prevalent in EP. The observed high rates of co-infection advocate the necessity of establishing national guidelines and/or screening program utilising multiplex PCR approach for the detection of common STIs among high risk groups in the kingdom. Further studies are needed to measure the adverse reproductive outcomes associated with STIs in Saudi Arabia.

## Background

Ectopic pregnancy (EP) is a pregnancy implanted outside the intrauterine cavity with over 98 % occurring within the Fallopian tube [1]. The prevalence of EP has been estimated to be 1-2 % of all natural pregnancies worldwide and in the kingdom of Saudi Arabia (KSA) it ranges between 0.58-1.5 % [2–5]. Tubal EP is the main cause of maternal mortality and morbidity during the first trimester and death secondary to EP represents 5 % and 10 % of all maternal deaths in developed and developing countries, respectively [6].

Several risk factors for the development of EP have been identified and a history of STI, tubal factor infertility (TFI) and/or pelvic inflammatory disease (PID) is particularly important and has been implicated in the pathogenesis of EP [7–9]. Damage to the lower and upper genital tracts can occur following bacterial, viral, fungal, and parasitic infection [10, 11]. PID refers to infection and inflammation of the upper genital tract (e.g. salpingitis and endometritis) in women and it leads to serious reproductive sequelae including infertility and EP [12–14]. A variety of pathogens have been identified in the aetiology of PID including *Chlamydia trachomatis* (CT), *Neisseria gonorrhoeae* (NG), *Mycoplasma genitalium* (MG), *Ureaplasma urealyticum* (Uurea), *Ureaplasma parvum* (Uparv), *Gardnerella vaginalis* (GV), *Trichomonas vaginalis* (TV) and herpes simplex virus (HSV) 1 & 2 [12–14].

STIs are major health problem and the World Health Organization (WHO) has estimated that 100 million persons acquire STIs every year and that 26 million people are infected in the Middle East [15]. Data on the frequency of STIs in Islamic countries is markedly limited and in KSA there has been no implementation of national guidelines and/or screening program to monitor the rate of STIs despite the recent findings of several studies from the kingdom [16–23]. Furthermore, the majority of these reports focused mainly on the prevalence of CT and NG and none of them measured the rate of other STIs and few were conducted on EP.

Several methods are available for detecting the aforementioned microorganisms in clinical specimens including culture, enzyme-linked immunosorbent assays and polymerase chain reaction (PCR) [24]. PCR has advantages over the other standard methods with respect to rapidity, sensitivity and specificity [24]. However, the microbiological aetiology of STIs involves multiple pathogens and therefore, multiplex PCR provides an additional advantage in screening since it detects multiple pathogens simultaneously in a single clinical sample with high sensitivity and specificity [25].

We hypothesise that STIs, similar to other societies, represent a significant risk factor for the development of EP in KSA. The current study was therefore designed to measure the prevalence of *C. trachomatis*, *N. gonorrhoeae*, *M. genitalium*, *U. urealyticum/parvum*, *G. vaginalis*, *T. vaginalis* and HSV-1/2 by multiplex TaqMan real-time PCR in Fallopian tube tissue samples collected from Saudi females diagnosed with EP and the results were compared with those obtained from women who underwent total abdominal hysterectomy (TAH) or tubal ligation (TL). The findings of this study may provide a better reflection on the role of STIs in the pathogenesis of EP and may possibly support the development of appropriate policies regarding screening and treatment of STIs especially for the healthcare policy makers in the kingdom.

## Methods

### Ethical approval

Ethical approval for the study was obtained from the Institutional Review Board of Umm Al-Qura university and Ethics Committee of the Faculty of Applied Medical Sciences (AMSEC 10-15-9-2011). All tubal samples were collected following obtaining informed written consent from all the participants.

### Study design

This was a prospective case–control study. Fallopian tube specimens were collected from 84 women diagnosed with tubal EP and they served as ‘Case group’. For the ‘Control group’, tubal specimens were collected from 31 patients during tubal ligation and 20 patients following total abdominal hysterectomy. The inclusion and exclusion criteria are summarised in Table 1. The study measured the frequency of *C. trachomatis*, *N. gonorrhoeae*, *M. genitalium*, *U. urealyticum/parvum*, *G. vaginalis*, *T. vaginalis* and HSV-1/2 by multiplex TaqMan real-time PCR in all samples and the results were compared between the case and control groups.

**Table 1:** Principle inclusion and exclusion criteria for the study groups

Control group		Case group	
Inclusion criteria	Exclusion criteria	Inclusion criteria	Exclusion criteria
Saudi	Non-Saudi	Saudi	Non-Saudi
Patient age ≥ 18 and ≤ 42 years.	Patient age < 18 or > 42 years.	Patient age ≥ 18 and < 40 years.	Patient age < 18 or > 40 years.
Cyclic women with a history of previous intrauterine pregnancy and no history of hydrosalpinx or ectopic pregnancy	Women with abnormal menstrual cycle, history of infertility treatment, history of PID, endometriosis and previous ectopic pregnancy.	Ectopic pregnancy following spontaneous conception	Ectopic pregnancy following assisted conception, using IUD, History/symptoms of urogenital infection (e.g. vaginal discharge, dysuria, PID, etc.)
Clinical decision to undertake total abdominal hysterectomy (TAH) for benign disease not affecting the Fallopian tubes and endometrium	Vaginal or subtotal hysterectomy	Singleton pregnancy	Multiple/heterotopic pregnancy
Clinical decision to undertake tubal ligation for sterilisation	Use of IUD ≤ 1 year prior to operation	Clinical determination that the patient is haemodynamically stable	Symptoms and signs of hypovolemia

### Study groups

A total of 135 participants were recruited from the Maternity and Children Hospital in Makkah (MH-M), Maternity hospital in Jeddah (MH-J) and Althager General Hospital in Jeddah (AH-J), KSA between January 2012 and February 2015.

- Ectopic pregnancy group (EP)
  Fallopian tube samples were collected from 84 women (mean age 32.7 ± 7.5 years) who attended the Emergency Department and/or the Early Pregnancy Assessment Unit with complaints of vaginal bleeding or lower abdominal pain during the first trimester of pregnancy. The diagnosis of EP was made either by transvaginal ultrasound or laparoscopy and salpingectomy was performed on clinical management grounds. The total numbers of patients recruited in this group from each centre during the 3 years of the study were 30 from the Maternity Hospital in Makkah (MH-M), 36 women from MH-J and 18 women form AH-J. Details of patient recruitment per year from each centre are summarised in Table 2.

**Table 2:** Details of recruitment of the 135 study participants according to study groups and study centres during the 3 years period of the study

	Maternity Hospital-Makkah				Maternity Hospital-Jeddah				Althagar General Hospital-Jeddah			
	*Year 1*	*Year 2*	*Year 3*	*Total*	*Year 1*	*Year 2*	*Year 3*	*Total*	*Year 1*	*Year 2*	*Year 3*	*Total*
*Ectopic pregnancy (n = 84)*	10	11	9	30	7	16	13	36	5	8	5	18
*Total Abdominal Hysterectomy ( n = 20)*	4	3	3	10	3	2	4	9	0	1	0	1
*Tubal Ligation (n = 31)*	5	5	3	13	4	5	4	13	2	3	0	5
*Total (n = 135)*	19	19	15	53	14	23	21	58	7	12	5	24
- Control group
  A set of 40 Fallopian tubes were obtained from 20 cycling women (mean age 37.2 ± 5.1 years) who were undergoing routine total abdominal hysterectomy for benign disease not affecting the Fallopian tubes. All women had regular menstrual cycles, were of proven fertility with no evidence of tubal disease and were not taking exogenous hormones (Table 2).

Another set of 62 tubal specimens were collected from 31 fertile women (mean age 36.1 ± 4.5 years) during tubal ligation for sterilisation at the time of caesarean section (Table 2). The collected tubes from both TAH and TL groups served as controls.

### Sampling and processing

All specimens were collected and processed under sterilised conditions. For the case group, the Fallopian tubes were excised and 1 cm from the implantation site was removed for routine histological analysis of the ectopic pregnancy. The remaining portions of the collected tubes were immediately cut using RNase/DNase-free equipment (baked at 200 °C for 4 h) into small pieces of 1 cm each. These samples were then transferred in 10 ml of sterile RNA*Later* solution (Ambion, Warrington, UK) for preservation. All the tissues were stored at −80 °C until processed for DNA extraction.

DNA was extracted from a random piece from each tubal specimen using a DNA extraction kit (Qiagen, CA, USA) according to the manufacturer’s instructions and following homogenisation using electrical homogeniser and sterile plastic probes (Omni International, GA, USA). The quality and quantity of extracted DNA were assessed with the BioSpec-nano (Shimadzu Corporation, Tokyo, Japan) and typically had an A260/A280 ratio of 1.7 to 1.9. Extracted DNA from all tubes were diluted to a final concentration of 50 ng/μl and aliquots of the diluted samples were stored at −20 °C until used.

Detection of human genomic DNA was performed by PCR using primers PCO3 and PCO6 specific for the human β-globin gene as previously described [26]. The expected PCR product of 326 bp was visualised on a 0.8 % agarose gel after ethidium bromide staining.

### Multiplex-PCR for 7 sexually transmitted pathogens in tubal tissues

Multiplex PCR amplification was performed on ABI® 7500 platform from Applied Biosystems (Thermo Fisher Scientific, Warrington, UK) and using the FTD STD9 kit (Fast-track diagnostics, Junglinster, Luxembourg) according to the manufacturer's protocol. The kit is IVD CE certified and contained sets of primers and TaqMan probes that were specifically designed from highly conserved regions of genetic sequences for the 7 pathogens (*C. trachomatis*, *N. gonorrhoeae*, *M. genitalium*, *U. urealyticum/parvum*, *G. vaginalis*, *T. vaginalis* and HSV-1/2). However, the kit does not distinguish either between the 2 *Ureaplasma* biovars or the HSV genotypes.

As reported by the manufacturer, the kit has a detection limit of 10^3^ copies/ml for all pathogens except TV and GV, which could be detected until 10^2^ copies/ml, and HSV1 that only could be measured until 10^4^ copies/ml. The analytical sensitivity of the kit also showed no loss of sensitivity of the FTD STD9 kit compared with singleplex assays as reported in the manufacturer validation document.

The FTD STD9 kit included murine cytomegalovirus as internal control and it was added to each Fallopian tubal sample at the lysis buffer stage of the extraction process, and co-amplified with the target DNA from all clinical samples to assure that the isolation of nucleic acid was successful and the absence of PCR inhibitors. The validation of the results was performed according to the manufacturer’s instructions and by using the provided positive and negative controls within the kit. Each well of the PCR plate contained 12.5 μl mastermix, 1 μl enzyme, 1.5 μl of primers and 10 μl (500 ng) DNA. The amplification was performed under the following conditions: 42 °C for 15 minutes hold, 94 °C for 3 minutes hold and 40 cycles (94 °C for 8 seconds and 60 °C for 34 seconds).

For those samples that were positive for NG, the results were validated by the FTD Gonorrhoeae confirmation kit (Fast-track diagnostics, Junglinster, Luxembourg) to confirm the results as per the manufacturer’s instructions and as previously described [27].

Since the kit is designed to be used with swab samples, culture, urine and thin prep samples and not with whole tissue, we performed an extra validation step for all samples that were negative to confirm the results. This step consisted of spiking the negative samples with the provided positive DNA controls at 4:1 ratio for a sample (8 μl) and positive control (2 μl), respectively. A detection of a signal following this step was reassuring that the designated organisms were not present in the original negative samples.

The determination of the gene copies/ml for each microorganism was carried out according the provided formula by the manufacturer that was based on the amplification dynamics of positive control dilution series with a linear relationship between log10 gene copy numbers and Ct values.

### Statistical analysis

Statistical analysis of the results was performed using SPSS version 16. Cross-tabulation followed by Chi square (X^2^) test or Fischer’s exact test were used for frequency analysis as appropriate, and a crude odds ratio (COR) with 95 % confidence interval (CI) were calculated. Binary regression was applied to measure the odds ratio (OR) and CI of each microorganism and single/multiple infection on the development of EP. All tests performed were two-sided and *P* value < 0.05 was considered significant.

## Results

### Subject characteristics

Between January 2012 and February 2015 a total of 135 women were recruited from 3 centres: 2 in Jeddah and the third from Makkah. The patients were 84 (62.23 %) women diagnosed with tubal ectopic pregnancy and who underwent salpingectomy, 20 women (14.81 %) who underwent routine total abdominal hysterectomy for benign diseases not affecting the tubes/endometrium and 31 patients (22.96 %) who had tubal ligation for sterilisation.

For the EP group, 35.7 % of cases (n = 30) were recruited from Makkah and the remaining 64.3 % (n = 54) were from Jeddah city. The cases of TAH were equally distributed between both cities (n = 10/city). On the other hand, 41.9 % (n = 13) of TL cases were recruited from Makkah and the remainder (n = 18) were from Jeddah. Details of the numbers of recruited patients from each study centre are summarised in Table 2.

There was a significant difference in the age of the case group (mean age 32.7 ± 7.5 years; range 26–38 years) when compared with the TAH group (mean age 37.2 ± 5.1 years; range 34–42 years) (*P* < 0.05). However, no significant difference was detected between TL group (mean age 36.1 ± 4.5 years; range 33–41 years) either with EP or TAH groups. Furthermore, there was no significant difference in the age between EP and control (TAH + TL; n = 51; *P* = 0.1).

### Prevalence of sexually transmitted infections

#### A. Overall prevalence

The M-PCR assay clearly distinguished and identified all seven STIs in tubal samples from the case and control groups either as single or co-infections. Signals were also detected in all wells that included the provided positive controls. Furthermore, no signal was observed with the negative controls. Spiked negative tubal samples with the provided DNA of the pathogens of interest also showed a positive signal reassuring the observed results of this study.

The overall prevalence of detecting the microorganisms of interest either as single or co-infections in the 135 study participants was 31.85 % (n = 43). By further analysis, the rates in descending order were 19.3 % (n = 26) for *C. trachomatis*, 15.6 % (n = 21) for HSV-1/2, 14.1 % (n = 19) for *M. genitalium*, 9.6 % (n = 13) *U. urealyticum/parvum*, 5.2 % (n = 7) *G. vaginalis*, 4.44 % (n = 6) for *N. gonorrhoeae* and 2.96 % (n = 4) for *T. vaginalis*.

The frequencies of single infection and co-infections in the 135 study participants were 11.1 % (n = 15) and 20.7 % (n = 28), respectively. The latter group included 15 patients positive for 2 pathogens (53.6 %), 10 patients positive for 3 pathogens (35.7 %) and 3 patients positive for the 7 pathogens (10.7 %).

#### B. Prevalence of STIs in case and control groups

One or more organisms were detected in tubal specimens collected from 36 women (42.85 %) with an ectopic pregnancy (COR 4.71; 95 % CI: 1.91-11.6; *P* = 0.001) and the frequency was significantly higher compared with the control group (13.72 %; n = 7). The frequencies of each organism in the study groups as single and co-infections are summarised in Table 3.

**Table 3:** Distribution of positive cases of *C. trachomatis* (CT), *N. gonorrhoeae* (NG), *M. genitalium* (MG), *U. urealyticum/parvum* (U. Urea/Parv), *G. vaginalis* (GV), *T. vaginalis* (TV) and herpes simplex virus (HSV)-1/2 in ectopic pregnancy (EP) and control (total abdominal hysterectomy and tubal ligation) groups according to single and co-infections (ND = Not detected; * = *P* < 0.05 compared with control and ‡ = *P* < 0.05 compared with single infection)

		Control (n = 51)	EP (n = 84)	Total (n = 135)
CT	*Single infection*	1 (1.9 %)	6 (7.2 %)^*^	7 (5.2 %)
	*Co-infections*	2 (3.9 %)	17 (20.2 %)^*,‡^	19 (14.1 %)^**‡**^
	*Total*	3 (5.9 %)	23 (27.4 %)^*^	26 (19.2 %)
NG	*Single infection*	ND	ND	ND
	*Co-infections*	1 (1.9 %)	5 (5.9 %)	6 (4.5 %)
	*Total*	1 (1.9 %)	5 (5.9 %)	6 (4.5 %)
MG	*Single infection*	ND	3 (3.6 %)	3 (2.2 %)
	*Co-infections*	2 (3.9 %)	14 (16.7 %)^*,‡^	16 (11.8 %)^**‡**^
	*Total*	2 (3.9 %)	17 (20.2 %)^*^	19 (14.1 %)
U. Urea/Parv	*Single infection*	1 (1.9 %)	2 (2.4 %)	3 (69.2 %)
	*Co-infections*	3 (5.9 %)	7 (8.4 %)^**‡**^	10 (30.8 %)^**‡**^
	*Total*	4 (7.8 %)	9 (10.7 %)	13 (9.6 %)
GV	*Single infection*	ND	ND	ND
	*Co-infections*	1 (1.9 %)	6 (7.2 %)^*^	7 (5.2 %)
	*Total*	1 (1.9 %)	6 (7.2 %)^*^	7 (5.2 %)
TV	*Single infection*	ND	ND	ND
	*Co-infections*	ND	4 (4.7 %)	4 (2.9 %)
	*Total*	ND	4 (4.7 %)	4 (2.9 %)
HSV-1/2	*Single infection*	ND	1 (1.2 %)	1 (0.75 %)
	*Co-infections*	3 (5.9 %)	17 (20.2 %)^*,‡^	20 (14.8 %)^**‡**^
	*Total*	3 (5.9 %)	18 (21.4 %)^*^	21 (15.6 %)

There was a significant difference between ectopic and control groups in the frequencies of *C. trachomatis* (COR 6.03; 95 % CI: 3.8-21.3; *P* = 0.001); *M. genitalium* (COR 6.2; 95 % CI: 2.3-18.1; *P* = 0.009) and HSV-1/2 (COR 4.3; 95 % CI: 2.2 – 15.6 %; *P* = 0.01). No significant difference was observed in the rates of the other microorganisms between the two groups (*P* > 0.05).

The presence of an infection (single/co-infection) was associated with a greater risk (COR 4.71; 95 % CI: 1.9 – 9.6; *P* = 0.0001) of developing EP. The rates of single infection (15.5 % vs. 3.9 %; COR 4.48; 95 % CI: 2.4 – 10.7; *P* = 0.02) and co-infections (27.4 % vs. 9.8 %; COR 3.5; 95 % CI: 1.22 – 8.8; *P* = 0.01) were significantly higher in EP group compared with control. Nevertheless, *N. gonorrhoeae*, *G. vaginalis* and *T. vaginalis* were not detected as a single infection in both the case and control groups (Table 3). Furthermore, *M. genitalium* and HSV-1/2 were only detected as mono-infection in the ectopic group.

By comparing between Makkah (single centre) and Jeddah (2 centres) cities, there was no significant difference in the prevalence of the candidate pathogens between the two cities except for *G. vaginalis*, which showed a significantly higher rates in those patients with EP and recruited from Makkah (Table 4). Additionally the frequencies of *C. trachomatis*, *M. genitalium* and HSV-1/2 were significantly higher in the EP group compared with control in both cities. Additionally, the rates of detecting each organism of interest did not vary significantly during the 3 years of the study period (data not shown). Although the results of copies/ml for the organisms of interest were higher in the EP group compared with control, we were not able to compute statistical analysis since the numbers of positive cases in the control group were very small (Table 5).

**Table 4:** Distribution of positive cases of *C. trachomatis* (CT), *N. gonorrhoeae* (NG), *M. genitalium* (MG), *U. urealyticum/parvum* (U. Urea/Parv), *G. vaginalis* (GV), *T. vaginalis* (TV) and herpes simplex virus (HSV)-1/2 in ectopic pregnancy (EP) and control (total abdominal hysterectomy and tubal ligation) groups according to single/co-infections and the city of recruitment (ND = Not detected; a = *P* < 0.05 compared to control from Makkah city, b = *P* < 0.05 compared to EP from Makkah centre, c = *P* < 0.05 compared with control from Jeddah city and * = *P* < 0.05 compared with single infection)

		Makkah centre		Jeddah centres	
		*Control (n = 23)*	*EP (n = 30)*	*Control (n = 28)*	*EP (n = 54)*
CT	*Single infection*	1 (4.3 %)	2 (6.7 %)	ND	4 (7.4 %)
	*Co-infections*	1 (4.3 %)	7 (23.3 %)^a,*^	1 (3.6 %)	10 (18.5 %)^c,*^
	*Total*	*2 (8.7 %)*	*9 (30 %)* ^*a*^	*1 (3.6 %)*	*14 (25.9 %)* ^*c*^
NG	*Single infection*	ND	ND	ND	ND
	*Co-infections*	ND	1 (4.3 %)	1 (3.6 %)	4 (7.4 %)
	*Total*	*ND*	*1 (4.3 %)*	*1 (3.6 %)*	*4 (7.4 %)*
MG	*Single infection*	ND	1 (4.3 %)	ND	2 (3.7 %)
	*Co-infections*	1 (4.3 %)	5 (16.7 %)^a,*^	1 (3.6 %)	9 (16.7 %)^c,*^
	*Total*	*1 (4.3 %)*	*6 (20 %)* ^*a*^	*1 (3.6 %)*	*11 (20.4 %)* ^*c*^
U. Urea/Parv	*Single infection*	ND	1 (3.3 %)	1 (3.6 %)	1 (1.9 %)
	*Co-infections*	1 (4.3 %)	2 (6.6 %)	2 (7.2 %)	5 (9.2 %)^*^
	*Total*	*1 (4.3 %)*	*3 (10 %)*	*3 (10.7 %)*	*6 (11.1 %)*
GV	*Single infection*	ND	ND	ND	ND
	*Co-infections*	ND	3 (10 %)	1 (3.6 %)	3 (5.6 %)
	*Total*	*ND*	*3 (10 %)* ^a,*^	*1 (3.6 %)*	*3 (5.6 %)* ^*b*^
TV	*Single infection*	ND	ND	ND	ND
	*Co-infections*	ND	1 (3.3 %)	ND	3 (5.6 %)
	*Total*	*ND*	*1 (3.3 %)*	*ND*	*3 (5.6 %)*
HSV-1/2	*Single infection*	ND	ND	ND	1 (1.9 %)
	*Co-infections*	1 (4.3 %)	6 (20 %)^a,*^	2 (7.1 %)	11 (20.4 %)
	*Total*	*1 (4.3 %)*	*6 (20 %)* ^*a*^	*2 (7.1 %)*	*12 (22.2 %)* ^*c*^

**Table 5:** The values of gene copies/ml of positive cases of *C. trachomatis* (CT), *N. gonorrhoeae* (NG), *M. genitalium* (MG), *U. urealyticum/parvum* (U. Urea/Parv), *G. vaginalis* (GV), *T. vaginalis* (TV) and herpes simplex virus (HSV)-1/2 in ectopic pregnancy (EP) and control (total abdominal hysterectomy and tubal ligation) groups according to single and co-infections (n = number of positive cases and ND = Not detected)

		Control	EP
CT (copies/ml)	*Single infection*	*(n = 1)*	*(n = 6)*
		6.06 X10^6^	2.45 X 10^7^ ± 1.43 X 10^5^
	*Co-infections*	*(n = 2)*	*( n = 17)*
		4.19 X 10^6^ ± 2.98 X 10^6^	4.22 X10^7^ ± 2.1 X10^7^
NG (copies/ml)	*Single infection*	*ND*	*ND*
	*Co-infections*	*(n = 1)*	*(n = 5)*
		6.24 X 10^4^	7.9 X 10^5^ ± 2.08 X 10^5^
MG (copies/ml)	*Single infection*	*ND*	*(n = 2)* 5.23 X 10^6^ ± 2.13 X10^6^
	*Co-infections*	*(n = 2)*	*(n = 14)*
		3.1 X 10^6^ ± 1.98 X10^5^	9.67 X 10^6^ ± 3.35 X10^7^
U. Urea/Parv (copies/ml)	*Single infection*	*(n = 1)*	*(n = 2)*
		2.2 X10^7^	7.54 X10^7^ ± 3.54 X 10^7^
	*Co-infections*	*(n = 3)*	*(n = 7)*
		1.58 X10^7^ ± 0.88 X10^7^	9.87 X10^7^ ± 2.15 X 10^7^
GV (copies/ml)	*Single infection*	*ND*	*ND*
	*Co-infections*	*(n = 1)*	*(n = 6)*
		3.89 X10^6^	11.95 X10^7^ ± 5.38 X 10^7^
TV (copies/ml)	*Single infection*	*ND*	*ND*
	*Co-infections*	*ND*	*(n = 3)*
			1.38 X10^6^ ± 0.71 X 10^6^
HSV-1/2 (copies/ml)	*Single infection*	*ND*	*(n = 1)*
			3.05 X10^4^
	*Co-infections*	*(n = 3)*	*(n = 17)*
		3.34 X10^4^ ± 1.41 X10^4^	2.90 X10^5^ ± 9.41 X10^4^

The results of binary logistic regression showed that infection with CT (OR 3.07; 95 % CI: 1.3 – 12.3; *P* = 0.002), MG (OR 2.3; 95 % CI: 1.1 – 8.6; *P* = 0.03), HSV-1/2 (OR 1.7; 95 % CI: 0.75 – 5.7; *P* = 0.004) and multiple infections (≥ 2 pathogens; OR 4.9; 95 % CI: 2.2 – 11.6; *P* = 0.006) was associated with a significantly higher risk of EP. However, neither the other microorganisms nor the age of patients increased the risk of EP (data not shown).

## Discussion

Herein, we measured the prevalence of 7 sexually transmitted organisms (*C. trachomatis*, *N. gonorrhoeae*, *M. genitalium*, *U. urealyticum/parvum*, *G. vaginalis*, *T. vaginalis* and HSV-1/2) detected simultaneously by multiplex TaqMan real-time PCR in Fallopian tubal specimens collected from 84 Saudi women diagnosed with EP and the results were compared with 51 control tubes obtained from 20 total abdominal hysterectomies and 31 tubal ligations. The prevalence of co-infections was significantly higher compared with single infection in the study participants and it was associated with 5 times greater risk of developing EP. The most prevalent microorganisms detected in EP were *C. trachomatis, M. genitalium* and HSV-1/2 and they were also associated with higher risks of EP. Although the rates of the other pathogens were also higher in the EP group, they were not statistically different between the case and control groups.

Our results suggest that STIs are common in Saudi Arabia and infections with *C. trachomatis, M. genitalium* and HSV-1/2 are more frequent in Saudi women suffering from ectopic pregnancy. Furthermore, the majority of positive cases in the present study (20.7 %) were positive for 2 or more sexually transmitted organisms and multiple infections with ≥ 2 pathogens increased the risk of EP and hence the use of multiplex PCR for the screening of STIs could be useful, especially for patients with tubal damage.

STI is a worldwide health problem and the WHO has estimated that 26 million people are infected in the Middle East [15]. Upper genital tract infection with sexually transmitted pathogens is a major risk factor for the development of PID and EP. An estimated 10 %–20 % of women with STI could develop PID if their infection was not identified [28], and a history of PID is associated with a 7.5 times greater risk of developing EP following either natural or assisted conception [29–31]. Reports from KSA have demonstrated that a substantial proportion of patients (up to 50 %) diagnosed with EP had a history of PID and/or infertility [4, 32–34]. Nevertheless, none of the aforementioned studies examined the types and rates of sexually transmitted organisms in specimens collected from EP [5].

*C. trachomatis* is the most common sexually transmitted bacterial infection worldwide and it has been estimated that 30 % of women will develop PID and tubal damage following infection with CT [35–38]. Several other non-chlamydial/non-gonococcal microorganisms have also been detected and isolated from infected upper genital tract tissue specimens and implicated in the pathogenesis of EP. *M. genitalium* has been associated with non-chlamydial/non-gonococcal urethritis, cervicitis as well as endometritis, PID and tubal factor infertility in women [39–42]. Additionally, HSV-1/2 are common sexually transmitted viruses and they have been isolated from tissue specimens collected from female upper genital tract [43–48]. Infection with HSV has also been shown to be involved in the pathogenesis of EP [49, 50], and has been reported to increase the risk of acquiring common STIs [51, 52].

Our results are in agreement with the previous reports as they showed a higher significant rates of *C. trachomatis*, *M. genitalium* and HSV-1/2 in specimens obtained from EP. Additionally, positive cases for these microorganisms showed co-infection with other STIs organisms, supporting the notion that tubal damage and development of EP could be induced by a variety of sexually transmitted pathogens alongside with *C. trachomatis*. Our findings also suggest that infection with *C. trachomatis*, *M. genitalium* and HSV-1/2 frequently colonise the female upper genital tract and could play a more important role in the pathogenesis of EP in KSA. However, the used kit in the present study could not discriminate between type 1 and 2 HSV and hence further studies using specific primers for the differentiation between the 2 types of HSV are needed to measure their rates in tubal tissue collected from EP. Furthermore, more studies are needed to explore possible socio-demographic risk factors that could be associated with acquisition of these microorganisms (e.g. educational level, income, etc.) and also possible pathogenic mechanism(s) by which these STIs induce tubal damage and promote the development of EP.

Although several studies reported an association between *N. gonorrhoeae* [53, 54], *U. parvum/urealyticum* [55–61] and *G. vaginalis* [13, 62, 63] with PID and tubal pregnancy, the current study detected non-significant higher rates of these pathogens in the EP group. Currently, there is no report in the literature from KSA on the prevalence of the aforementioned pathogens. Although, we observed high rates of *U. parvum/urealyticum* and *G. vaginalis* in our study population, our data does not provide evidence whether these bacteria could be involved in adverse reproductive outcomes in Saudi women. Hence, additional studies with larger numbers of patients are needed to measure the role of these microorganisms in upper genital tract infections and poor reproductive performance in KSA. Additionally, commercial kits and/or an in house PCR technique should be used to differentiate between the biovars of ureaplasmas since the currently used kit does not distinguish between *U. parvum* and *U. urealyticum* since these microorganisms, especially GV and *U. parvum* are common in normal women.

*T. vaginalis* is the most common non-viral sexually transmitted infection with and estimated annual prevalence of 170 million new cases worldwide [14]. Although TV is associated with bacterial vaginosis and the subsequent development of PID [64, 65], currently there is no direct association between the protozoa and EP. However, several reports have previously demonstrated the isolation of TV from pelvic organs and upper genital tract tissues [65–67]. The current findings are in agreement with the previous observations as we were able to detect TV in 4 tubal specimens collected from EP and none from the control group. However, there was no significant difference between the 2 groups in the frequency of positive cases. Additionally, all positive cases had multiple infections with other sexually transmitted pathogens, suggesting that TV could indirectly induce EP by promoting the acquisition of other STIs [68–70]. Further studies are required to explore the role(s) of TV in the pathogenesis of PID and tubal damage.

Information about STIs in Islamic countries generally and in KSA specifically is notably limited [18, 71]. Furthermore the majority of reports generated from Saudi Arabia focused on the frequency of *C. trachomatis* and *N. gonorrhoeae* and little is known about the other STIs included in our study. The first report by the Saudi Ministry of Health (MOH) on STIs between 1995–1999 has shown that among 39049 reported cases of STIs, the majority of patients were Saudi (70.4 %) and the prevalence among them was 84.1 % for non-gonococcal urethritis, 70.9 % for trichomoniasis, 77.1 % for gonococcal infection and 78 % for genital herpes. Furthermore, the estimated annual incidence was 14.8 for non-gonococcal infection, 9.4 for trichomoniasis, 5.2 for gonococcal urethritis and 0.1 for HSV per 100.000 population during the same period [71]. A more recent retrospective report that examined the records of the MOH during 2009 has also shown that STIs were more common in Saudi compared with non-Saudi and the majority of positive cases were females (92.2 %) during the reproductive age [18].

Studies that examined individual sexually transmitted pathogen have reported a prevalence of 4 to 21 % for chlamydial infection in asymptomatic pregnant women [16, 22, 72–75]. Similar results were also reported by Alzahrani et al. (2010) and they showed that 10.5 % of 95 healthy pregnant women were positive for *C. trachomatis* by serology and culture*.* However, the prevalence of chlamydial infection in 102 female patients attending the gynaecology clinic of the same hospital with symptoms of lower genital tract infection was 34.4 % [16]. Additionally, more recent studies conducted among infertile women in the kingdom have reported *C. trachomatis* in 9.6 % by serology and 12.03 % by culture method [22] and, the frequency ranges between 8-25 % by PCR [17, 21, 76].

The published reports on the prevalence of non-chlamydial sexually transmitted pathogens in KSA are few. The rate of *N. gonorrhoeae* was 7.8 % among women with symptoms of lower genital tract infection [16]. Previous studies also demonstrated that the seroprevalence of HSV-1 among adult Saudi was 60 % using indirect immunoassay [77, 78]. Later, Ghazi et al. (2002) reported a seroprevalence of 90.9 % for HSV-1 and 27.1 % for HSV-2 in normal pregnant women [79]. A more recent study showed that the frequency of HSV-1 and HSV-2 among 459 pregnant Saudi women was 84.1 % and 6.8 %, respectively [80]. Furthermore, Fageeh (2009) has reported an overall frequency of 74.5 % for HSVs in pregnant women attending to the emergency ward and among the positive HSV cases, IgG antibodies against CMV, *C. trachomatis* and *N. gonorrhoeae* were detected in 11.3 %, 12.8 % and 4.7 %, respectively [23].

Recently, we have shown that the prevalence of *C. trachomatis* IgG, but not IgM, was significantly higher in Saudi patients with EP (18.5 %) compared with normal pregnancy (5 %). Additionally, the frequency of HSV-1 IgG, but not HSV-2, was also higher in ectopic pregnancy (94.3 %) than in normal pregnancy (64 %) and the rate of detecting both IgM and IgG antibodies together either against HSV-1 or HSV-2 was higher in the ectopic (41.1 % and 30 %, respectively) compared to control (9 % and 16 %, respectively) [81].

Our results are in agreement with the previous studies conducted in the kingdom and they showed an overall prevalence of 19.25 % for chlamydia infection. The frequency was 27.4 % for EP, which is comparable to those reported from patients with infertility and urogenital symptoms [16, 17, 76]. Additionally, positive cases for HSV-1/2 DNA represented 15.6 % of the study population and the rates were also significantly higher in the EP group (21.4 %) compared with control (5.9 %). The overall rate of gonococcal infection in this report was 4.44 % and there was no significant difference in the frequency of positive cases between the EP (5.9 %) and control (1.9 %) groups. Our observations provide further support for the recent call to implement a national screening program for the detection of STIs in KSA [5, 16, 21, 76].

A drawback to our study is that we did not include other urogenital specimens (e.g. first void urine) and/or not performing serological studies, which would reflect on the prevalence of lower genital tract infection and previous exposure to the pathogens of interest in our study population. Nevertheless, the currently used samples are more appropriate to provide a better reflection on the rate of upper genital tract infection, which is aligned with our study objectives. Additionally, we did not confirm the results of this study by a different technique and/or other well-established commercial PCR kits. However, the used kit is IVD CE certified and we further performed extra steps to validate the observed results. Nevertheless, future studies should consider the inclusion of other commercial/in-house PCR protocols or confirm the results with additional laboratory techniques.

A further limitation is that we were not able to identify other confounding factors that could have a role in the observed increase in the prevalence of STIs among Saudi women diagnosed with EP (e.g. number of sexual partners, socio-economic factors, etc.), which was mainly due to social and culture restrictions since discussion of STIs in the Saudi community is considered taboo due to ethics and many social factors that create several obstacles [5, 16, 17, 19, 21].

## Conclusions

Infection of the upper genital tract with STIs is common in Saudi women from the Western region during the reproductive age and the prevalence of *C. trachomatis*, *M. genitalium* and HSV-1/2 was significantly higher in the EP group. Hence, future studies on the effect of these microorganisms on tubal ciliary beat frequency and the expression of implantation markers by the tubal epithelium are required to establish whether they play a role in the pathogenesis of EP. Additionally, the observed high rates of co-infection advocate the necessity of establishing national guidelines and/or screening program in the kingdom that could probably adopt syndromic approach using multiplex PCR technique for the simultaneous detection of the common sexually transmitted pathogens among high risk groups. Further studies are needed to explore the potential socio-economic risk factors associated with the acquisition of STIs and to measure their secondary adverse reproductive outcomes in Saudi Arabia.

## Abbreviations

AH-J: Althager General Hospital in Jeddah
bp: Base pair
CI: Confidence interval
COR: Crude odds ratio
CT: *Chlamydia trachomatis*
EP: Ectopic pregnancy
GV: *Gardnerella vaginalis*
HSV: Herpes simplex virus
KSA: Kingdom of Saudi Arabia
MH-J: Maternity hospital in Jeddah
MH-M: Maternity and Children Hospital in Makkah
MG: *Mycoplasma genitalium*
M-PCR: Multiplex Polymerase chain reaction
MOH: Ministry of Health
NG: *Neisseria gonorrhoeae*
OR: Odds ratio
PCR: Polymerase chain reaction
PID: Pelvic inflammatory disease
STI: Sexually transmitted infection
TAH: Total abdominal hysterectomy
TFI: Tubal factor infertility
TL: Tubal ligation
Uurea: *Ureaplasma urealyticum*
Uparv: *Ureaplasma parvum*
WHO: World Health Organization
